# High-Throughput Screening of a 2-Keto-L-Gulonic Acid-Producing *Gluconobacter oxydans* Strain Based on Related Dehydrogenases

**DOI:** 10.3389/fbioe.2019.00385

**Published:** 2019-12-13

**Authors:** Yue Chen, Li Liu, Xiaoyu Shan, Guocheng Du, Jingwen Zhou, Jian Chen

**Affiliations:** ^1^Key Laboratory of Industrial Biotechnology, Ministry of Education and School of Biotechnology, Jiangnan University, Wuxi, China; ^2^National Engineering Laboratory for Cereal Fermentation Technology, Jiangnan University, Wuxi, China; ^3^Jiangsu Provisional Research Center for Bioactive Product Processing Technology, Jiangnan University, Wuxi, China; ^4^The Key Laboratory of Carbohydrate Chemistry and Biotechnology, Ministry of Education, School of Biotechnology, Jiangnan University, Wuxi, China

**Keywords:** 2-keto-L-gulonic acid, L-sorbose, 2-ketoaldonate reductase, high-throughput screening, fluorescence-activated cell sorting, *Gluconobacter oxydans*

## Abstract

High-throughput screening is a powerful tool for discovering strains in the natural environment that may be suitable for target production. Herein, a novel enzyme-based high-throughput screening method was developed for rapid screening of strains overproducing 2-keto-L-gulonic acid (2-KLG). The screening method detects changes in the fluorescence of reduced nicotinamide adenine dinucleotide (NADH) at 340 nm using a microplate reader when 2-KLG is degraded by 2-KLG reductase. In this research, three different 2-KLG reductases were expressed, purified, and studied. The 2-KLG reductase from *Aspergillus niger* were selected as the best appropriate reductase to establishment the method for its high activity below pH 7. Using the established method, and coupled with fluorescence-activated cell sorting, we achieved a high 2-KLG-producing strain of *Gluconobacter oxydans* WSH-004 from soil. When cultured with D-sorbitol as the substrate, the 2-KLG yield was 2.5 g/L from 50 g/L D-sorbitol without any side products. Compared with other reported screening methods, our enzyme-based method is more efficient and accurate for obtaining high-producing 2-KLG strains, and it is also convenient and cost-effective. The method is broadly applicable for screening keto acids and other products that can be oxidized via nicotinamide adenine dinucleotide (NAD^+^) or nicotinamide adenine dinucleotide phosphate (NADP^+^).

## Introduction

2-Keto-L-gulonic acid (2-KLG) is the direct precursor for the industrial synthesis of vitamin C (Gao et al., 2014), and it can also make L-xylose, the precursor of L-xylitol (Zhang et al., 2017). Both vitamin C and L-xylitol are important products used extensively in food, beverage and pharmaceutical industries (Zou et al., 2013; Ur-Rehman et al., 2015). Currently, 2-KLG is mainly prepared by a two-step fermentation process, which utilizes *Gluconobacter oxydans* to convert D-sorbitol to L-sorbose, combined with mixed fermentation of *Ketogulonicigenium vulgare* and *Bacillus megaterium* to convert L-sorbose to 2-KLG (Takagi et al., 2010). The mechanism of 2-KLG production is intricate and still not fully understood despite much research on the relationship between *K. vulgare* and *B. megaterium* (Ma et al., 2011; Zhou et al., 2011; Zhu et al., 2012; Ding et al., 2014; Fan et al., 2014; Ye et al., 2014; Mandlaa et al., 2015; Pan et al., 2017). Random mutagenesis and selection are the main strategies for obtaining high-efficiency mutants (Yang et al., 2017), but there are only a few successful studies on molecular reconstitution of *K. vulgare* to enhance 2-KLG production (Cai et al., 2012; Du et al., 2013).

Thus, screening large numbers of mutants is a major challenge. However, iodimetric and high-performance liquid chromatography (HPLC) methods are currently the principal assays for identifying 2-KLG (Choi et al., 1996), and both are cumbersome and time-consuming. Various screening methods have been developed for the detection of target products, including colorimetric methods in which a substrate or product changes color (Nakamura et al., 2001; Furubayashi et al., 2014), pH-based strategies when the pH changes (Persson and Palcic, 2008; Zeng et al., 2015), and fluorescence-based enzyme-coupled strategies when a fluorescent substance is consumed or generated (Reetz, 2002). A pH-based plate method for rapid screening *K. vulgare* mutants was recently reported, but this method cannot differentiate 2-KLG from other acidic metabolites in the medium, and it cannot accurately probe the fermentation progress (Yang et al., 2017). An enzyme-coupled screening method would be more advantageous due to higher substrate specificity and direct correlation with fermentation, rather than indirect pH control.

2-KLG can be degraded by many microorganisms such as *Escherichia coli, Pseudomonas putida, Erwinia sp*., and *Aspergillus niger* (Makover et al., 1975; Truesdell et al., 1991; Yum et al., 1998; Kuivanen et al., 2017). In this process, reductases convert 2-KLG to L-idonic acid (Yum et al., 1998; Kuivanen et al., 2017), dehydrogenases act on 2-KLG to generate 2,5-diketo-D-gluconic acid (Miller et al., 1987), and decarboxylases transform 2-KLG into L-xylose (Stribny et al., 2016). Among them, 2-ketoaldonate reductase (2-KLG reductase) consumes two molecules of NAD(P)H to form NAD(P)+ when it degrades one molecule of 2-KLG. Microplate readers detect changes of absorbance at 340 nm when NAD(P)H increases or decreases.

Herein, we established a high-throughput screening method for 2-KLG using heterologous expression of different 2-KLG reductases in *Escherichia coli* BL21 and purifying them by Nickel-nitrilotriacetic acid (Ni-NTA) affinity chromatography. This method can rapidly and precisely determine the concentration of 2-KLG in the fermentation broth. Three different 2-KLG reductases were tested, and 2-KLG reductase (GLUD) from *Aspergillus niger* (Kuivanen et al., 2017) was chosen for the high-throughput screening method due to its high activity below pH 7, better stability between 35 and 40°C, and most importantly, a higher V_max_ value with the 2-KLG substrate. This high-throughput screening method can measure the concentration of 2-KLG in the media hundreds of times faster than HPLC with a comparable accuracy. Employing this method, we obtained a *G. oxydans* WSH-004 strain from soil that achieved the *de novo* production of 2-KLG. Additionally, this method can be applied to the screening of *K. vulgare* in mixed cultures, and for screening other metabolic products that consume or produce NAD(P)H.

## Materials and Methods

### Genes, Plasmids, and Strains

The wild type strain *Gluconobacter oxydans* WSH-004 (CCTCC M2019481) was screened from the soil in this study. Strain *E. coli* JM109 was used for plasmid construction. Strain *E. coli* BL21 (DE3) and vector pET28a(+) were used for protein expression. The *2kr* gene of *Citrobacter freundii* (GenBank: JMTA01000002.1) was amplified with primer pair *cfr-2kr-Eco*RI-F (5′-CCGGAATTCATGAAGCCGTCCATTATTCTCTATAAAGCG-3′) and *cfr-2kr-Sal*I-R (5′-ACGCGTCGACTCGCGCGACCTGCGGGTTTAC-3′) (restriction enzyme sites were underlined), subcloned into the T-vector pMD19, and verified by Sanger sequencing (Sangon Biotech, Shanghai, China). The termination codons of the *2kr* gene of *Aspergillus niger* (Gene ID: 4977968) and the *GluD* gene of *Aspergillus niger* (GenBank: KX443112.1) were removed, *Bam*HI (GGATCC), and *Sal*I (GTCGAC) restriction enzyme sites were introduced at the beginning and end, respectively, and genes were synthesized by Huada (Beijing Liuhe Huada Gene Technology Company, Beijing, China). These genes were inserted into plasmid pET28a(+) by standard double restriction enzyme digestion and ligation procedures. Recombinant plasmids were transformed into *E. coli* BL21 (DE3) cells for protein expression. All plasmids and strains are listed in Table 1.

**Table 1:** Plasmids and strains used in this study.

**Plasmids or strains**	**Characteristics**	**Sources**
**Plasmids**		
pET-cfr2kr	pET28a(+) carrying *2kr* from *citrobacter freundii* ATCC 8090f	This study
pET-ani2kr	pET28a(+) carrying *2kr* from *Aspergillus niger* CBS 513.88	This study
pET-anigluD	pET28a(+) carrying *gluD* from *Aspergillus niger* ATCC 1015	This study
**Strains**		
WSH-004	High-producing 2-KLG strain	This study
str-control	*E. coli* BL21 (DE3) carrying pET28a(+)	This study
str-cfr2kr	*E. coli* BL21 (DE3) carrying pET-cfr2kr	This study
str-ani2kr	*E. coli* BL21 (DE3) carrying pET-ani2kr	This study
str-anigluD	*E. coli* BL21 (DE3) carrying pET-anigluD	This study

### Media and Culture Condition

Luria-Bertani (LB) medium (10.0 g peptone, 5.0 g yeast extract and 10.0 g sodium chloride per L) was used to culture *E. coli* cells. Terrific Broth (TB) medium (12.0 g peptone, 24.0 g yeast extract, 4 mL glycerol, 2.31 g KH_2_PO_4_ and 16.42 g K_2_HPO_4_·3H_2_O per L) was used for protein expression. Sorbitol/sorbose medium (10 g sorbitol, 10 g sorbose and 5 g yeast per L) was used to screen 2-KLG-producing strains at 28°C with shaking at 220 rpm. Sorbitol medium (50 g sorbitol and 10 g yeast per L) was used to culture *Gluconobacter oxydans* at 30°C with shaking at 220 rpm.

### Enzyme Expression and Purification

Recombinant strains were inoculated into LB medium in a 14 mL round-bottomed tube overnight at 37°C with shaking at 220 rpm. Recombinant strains were cultured in 250 mL shake flasks with 25 mL TB medium to 1% (v/v), 500 μM isopropyl-β-D-1-thiogalactopyranoside (IPTG) was added to induce protein expression when cells had reached log phase, and culturing was continued at 25°C for a further 8 h at 25°C. Cells were washed twice, resuspended in 50 mM phosphate-buffered saline (PBS; 50 mM NaH_2_PO_4_-Na_2_HPO_4_, pH 7.0) and subjected to ultrasonication on ice. The supernatant was used to determine protein concentration and expression after centrifugation. Protein concentration was determined using a Bradford protein assay kit (Beyotime, Nantong, China). Recombinant protein expression was determined by sodium dodecyl sulfate polyacrylamide gel electrophoresis (SDS-PAGE) and Coomassie Brilliant Blue staining. *E. coli* BL21 (DE3) cells harboring empty pET28a(+) were used as a control. Ampicillin (100 μg/mL) and kanamycin (50 μg/mL) were used where required.

All 2-KLG reductase genes were fused with a His-tag and proteins were purified by Ni-NTA resin. Supernatants were bound to Ni Sepharose and washed with five column volumes of binding buffer consisting of 50 mM phosphate buffer (PB), 150 mM NaCl and 25 mM imidazole. Enzymes were eluted with five column volumes of elution buffer (50 mM PB, 150 mM NaCl and 500 mM imidazole). Finally, enzymes were desalted using a gel column with desalting buffer (50 mM PB and 150 mM NaCl) to remove imidazole. All purification operations were performed at 4°C.

### Enzyme Assay of 2-KLG Reductases

One unit of 2-KLG reductase activity was defined as the amount degrading 1 μM NADH in 1 min at 37°C and pH 7.0. Reaction kinetics were determined based on consumption of NADH (the decrease in absorbance at 340 nm, A340). Activity of 2-KLG reductases was normalized by dividing by the respective protein concentration. The optimal pH and temperature were measured with reaction mixtures containing 50 mM buffer, 400 μM NADH or NADPH, 10 mM 2-KLG, and purified 2-KLG reductases at a final concentration of 40 mg/L. Different pH values were achieved using 50 mM sodium acetate-acetate (pH 4.0), 50 mM PBS (pH 5.0, 6.0, 7.0, 8.0, and 9.0) and 50 mM TRIS-HCl (pH 10.0). Activity was tested at different temperatures (25, 30, 35, 40, 45, 50, and 55°C) in 1.5 mL tubes. Assays were also conducted with different substrate concentrations and purified 2-KLG reductases at a final concentration of 0.5 mg/L. For HPLC analysis and liquid chromatography-ion trap-time of flight tandem mass spectrometric assay (LCMS-IT-TOF), mixtures contained 50 mM PBS, 10 mM NADH or NADPH, 10 mM substrate (2-KLG, D-sorbitol, L-sorbose, D-fructose or D-glucose), and purified 2-KLG reductases at a final concentration of 4 mg/L. All enzyme assays were carried out independently in triplicate.

### Methods for Screening

Strains from soil and red tea fungus were enriched at 28°C with shaking at 220 rpm in 250 mL shake flasks with 25 mL sorbitol/sorbose medium for 48 h. Cells were centrifuged and resuspended in 50 mM PBS. After treating with 10 μg/mL fluorescein diacetate for 20 min, live cells were separated into 48 deep-well multi-well plates (48-well MTPs) containing 1 mL of sorbitol/sorbose medium by fluorescence-activated cell sorting (FACS) (Cao et al., 2018) The 48-well MTPs were cultured for 5 days at 30°C and 220 rpm, then centrifuged for 10 min at 4,000 g in a deep-well multi-well plate centrifuge (Beckman Coulter, Brea, CA). Aliquots of supernatants (40 μL) were transferred to a 96-well enzyme-labeled plate for detection at 340 nm. High-producing strains were transferred to 250 mL shake flasks containing 25 mL sorbitol/sorbose medium and the concentration was verified by HPLC (Supplementary Figure 1).

### HPLC and Liquid Chromatography Ion Trap Time-of-Flight Mass Spectrometry (LCMS-IT-TOF) Assays

D-sorbitol, L-sorbose, 2-KLG, D-fructose and D-glucose were determined by HPLC using an Aminex HPX-87H column (Bio-Rad, Hercules, CA, USA) at 35°C with a flow rate of 0.5 mL/min and 5 mmol/L H_2_SO_4_ as the eluent. The product L-idonic acid was analyzed with a Shimadzu LCMS-IT-TOF (Shimadzu, Kyoto, Japan). An Aminex HPX-87H column was used to perform HPLC separation at 35°C with a flow rate of 0.5 mL/min and 5 mmol/L formic acid as the eluent. IT-TOF detection was performed with an ESI source in negative and positive ion mode at the followed conditions: detector voltage, 1.60 kV; nebulizing gas (N_2_) flow, 1.5 L/min; drying gas (N_2_) flow, 200 kPa; ion accumula- tion time, 30 ms; and scan range (m/ z), 100–300 for MS1 (Gao et al., 2014).

## Results

### Expression and Purification of 2-KLG Reductase

**Figure 1:**
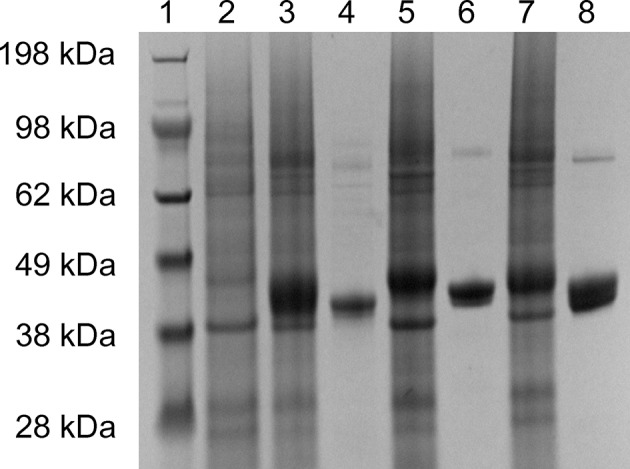
Expression and purification of 2-KLG reductase. Soluble proteins were used for verification of recombinant 2-KLG reductase by SDS-PAGE. Lane 1: molecular mass markers (Thermo Fisher Scientific); lane 2: str-control; lane 3: str-cfr2kr; lane 4: purified CFR-2KR; lane 5: str-ani2kr; lane 6: purified ANI-2KR; lane 7: str-anigluD; lane 8: purified ANI-GLUD.

**Figure 2:**
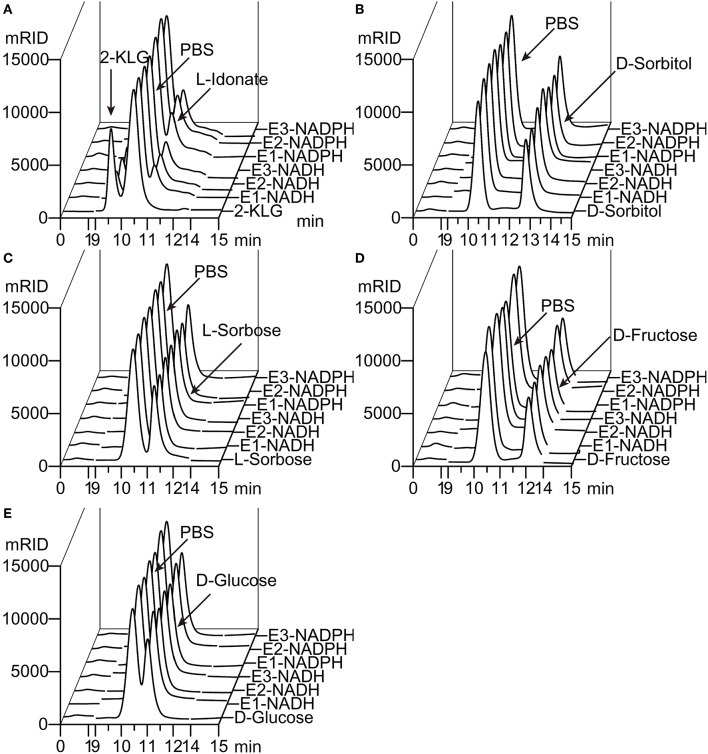
Substrate specificity of 2-KLG reductases. **(A–E)** Substrate 2-KLG, D-sorbitol, L-sorbose, D-fructose, and D-glucose were tested to verify the specificity of three 2-KLG reductases. E1, E2, and E3 stands for CFR-2KR, ANI-2KR and ANI-GLUD, respectively. NADH stands for reduced nicotinamide adenine dinucleotide and NADPH stands for reduced nicotinamide adenine dinucleotide phosphate. All the substrates were dissolved to 50 mM PBS (phosphate-buffered saline) as the control. The reaction mixtures contained 50 mM PBS, 10 mM NADH or NADPH, 10 mM substrate (2-KLG, D-sorbitol, L-sorbose, D-fructose or D-glucose), and purified 2-KLG reductase at a final concentration of 4 mg/L. The results were analyzed by HPLC.

The 2-KLG reductases were expressed by growing bacteria to OD_600_ ~0.6–0.8 at 37°C then inducing with 0.5 mM IPTG for 8 h at 25°C. His-tagged reductases were purified by metal affinity chromatography on a Ni-NTA column and verified by SDS-PAGE and enzyme activity assays. As shown in Figure 1, str-cfr2kr (CFR-2KR), str-ani2kr (ANI-2KR), and str-anigluD (ANI-GLUD) all yielded a single band following SDS-PAGE and staining with Coomassie Blue that corresponded with the calculated molecular weight of the protein deduced from the nucleotide sequence (41.2, 42.1, and 41.5 kDa, respectively). The purified reductases were further verified by enzyme activity assays and the product was detected by HPLC. All three 2-KLG reductases displayed enzyme activity with 2-KLG as substrate and NADH or NADPH as cofactor at pH 7.0 and 30°C (Figure 2).

### Enzymatic Properties of Different 2-KLG Reductases

**Figure 3:**
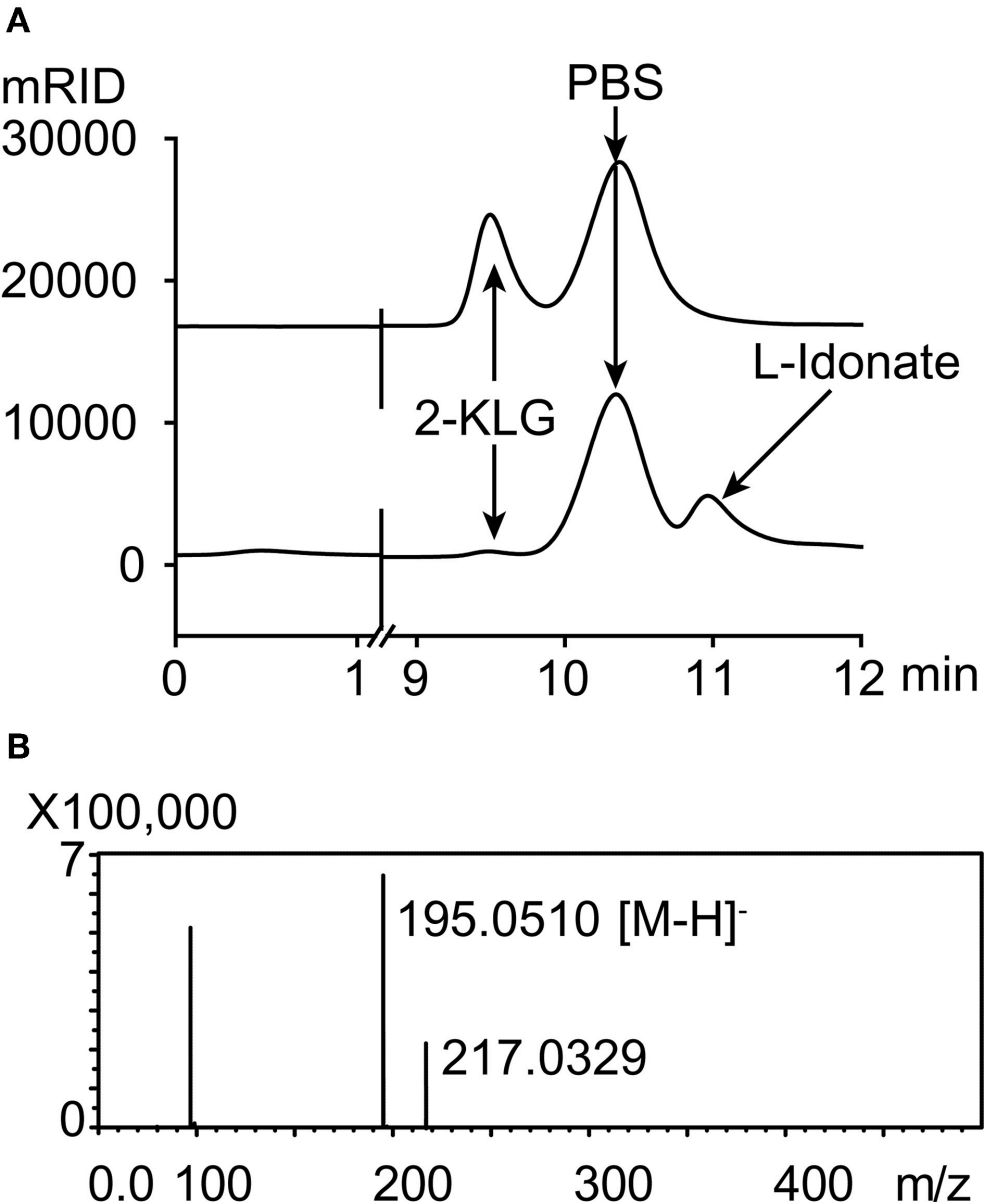
HPLC and mass spectrometry analyses of 2-KLG and L-idonate. **(A)** Determination of 2-KLG and L-idonate by HPLC. **(B)** Determination of L-idonate by LCMS-IT-TOF in negative ion mode.

Five different substrates were tested to explore the specificity of 2-KLG reductases. As shown in Figure 2, all three 2-KLG reductases displayed a high degree of specificity only reacted with 2-KLG but not D-sorbitol, L-sorbose, D-fructose or D-glucose, which are the main products of the sorbitol pathway. The molecular mass of the reduced product was 196 Da, consistent with the expected mass for L-idonate (Figure 3).

**Figure 4:**
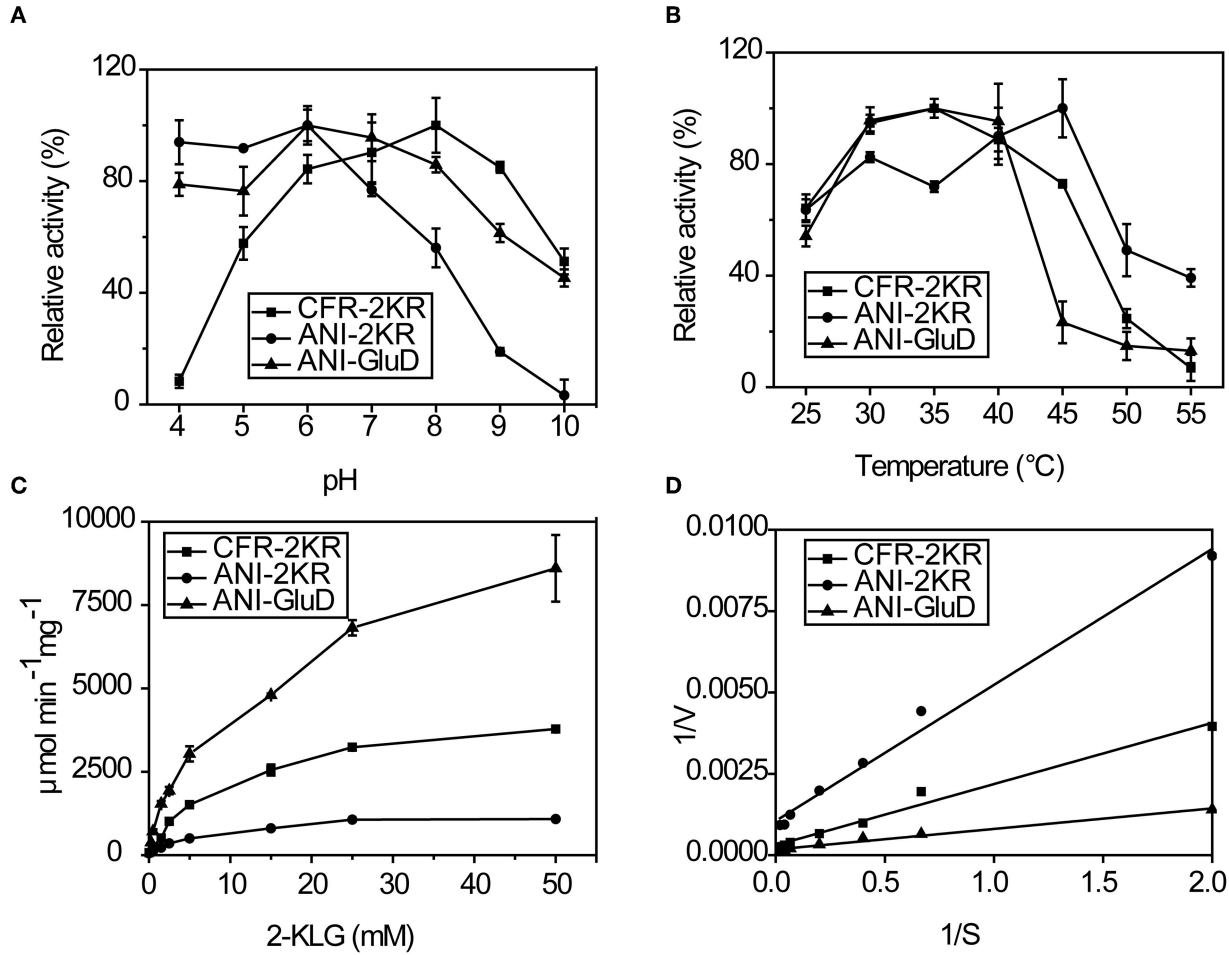
Enzymatic activity of recombinant 2-KLG reductases. **(A)** Effect of pH on 2-KLG reductases. **(B)** Effect of temperature on 2-KLG reductases. **(C)** Effect of substrate concentration on 2-KLG reductases. **(D)** K_m_ and V_max_ values for 2-KLG reductases.

The effects of pH, temperature and substrate concentration on 2-KLG reductases were investigated. CFR-2KR exhibited optimal activity pH at 8, while ANI-2KR and ANI-GLUD were most active at pH 6. Both ANI-2KR and ANI-GLUD had high activity at low pH, while CFR-2KR lost most of its activity below pH 4 (Figure 4A). Both CFR-2KR and ANI-GLUD displayed highest activity at 35°C, compared with 45°C for ANI-2KR (Figure 4B). Among the three reductases, ANI-GLUD exhibited the highest V_max_ value (8.7 mmol/min/mg), followed by ANI-2KR (4.0 mmol/min/mg) and CFR-2KR (1.2 mmol/min/mg; Figures 4C,D).

### Establishing an Enzyme-Based High-Throughput Screening Method

There are some important considerations when attempting to establish and enzyme-based high-throughput screening method. Firstly, the high substrate specificity of 2-KLG reductase is important for precise measurement of the 2-KLG concentration in the fermentation broth. Additionally, because the fermentation broth is acidic and contains various organic acids including 2-KLG ring the latter stages of fermentation, high and stable activity at low pH is preferable. Moreover, a reductase with high activity at low temperatures is desirable because enzymes tend to be more stable at lower temperatures. Finally, a high V_max_ value is of course desirable. Thus, ANI-GLUD was chosen as the best 2-KLG reductase for establishing a high-throughput screening method due to its high substrate specificity, high and stable activity at low pH and low temperature, and highest V_max_ value among the three reductases tested.

**Figure 5:**
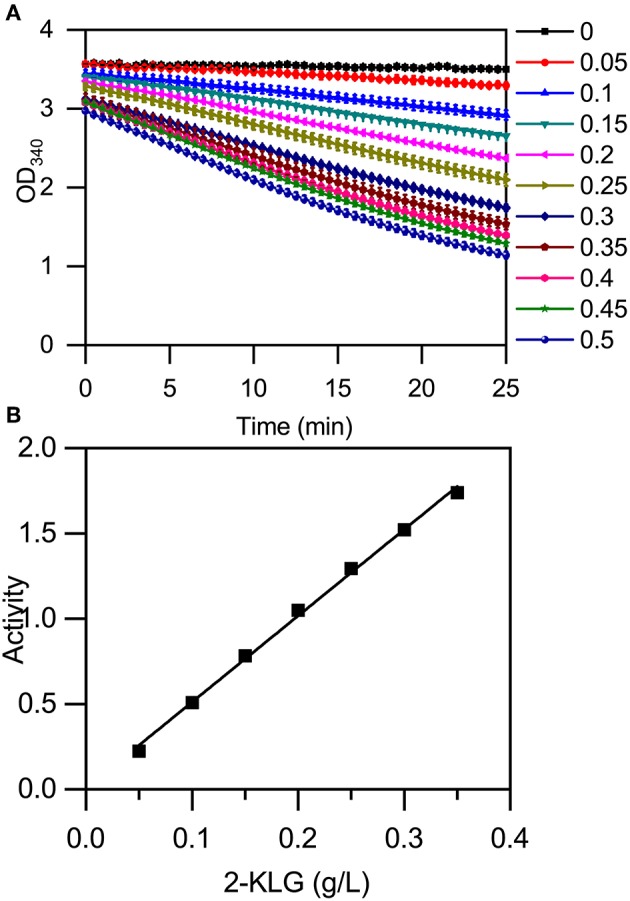
Establishment of an enzyme-based high-throughput screening method. **(A)** Microplate reader results using 2-KLG standards (dissolved in 50 mM PBS at a final concentration from 0 g/L to 0.5 g/L). **(B)** The high-throughput screening method has an excellent linear detection range for 2-KLG concentrations between 0.05 and 0.35 g/L.

The high-throughput screening method was established using a microplate reader to detect the change in absorbance at 340 nm that accompanies a change in the concentration of NADH (Figure 5A). The reaction mixture contained 50 mM buffer solution, 400 μM NADH, and purified 2-KLG reductases at a final concentration of 40 mg/L. Standards or samples were added to reaction mixtures in 96-well enzyme-labeled plates before the reaction mixtures. Reaction mixtures were assessed using the microplate reader. As shown in Figure 5A, the slope represents the concentration of 2-KLG and the greater the slope, the higher the concentration. All detection processes for 96 samples could be detected simultaneously within 15 min, while it took 20 min for one single sample by HPLC. Compared with the method of HPLC, the enzyme based method was about 100-fold faster. Most importantly, this method measured the concentration of 2-KLG precisely with an error <0.01 g/L (verified by HPLC, data not shown) and displayed an excellent linear detection range for 2-KLG between 0.05 and 0.35 g/L with and R-value of 0.997 (Figure 5B).

### Identification of High-Producing 2-KLG Strains

**Figure 6:**
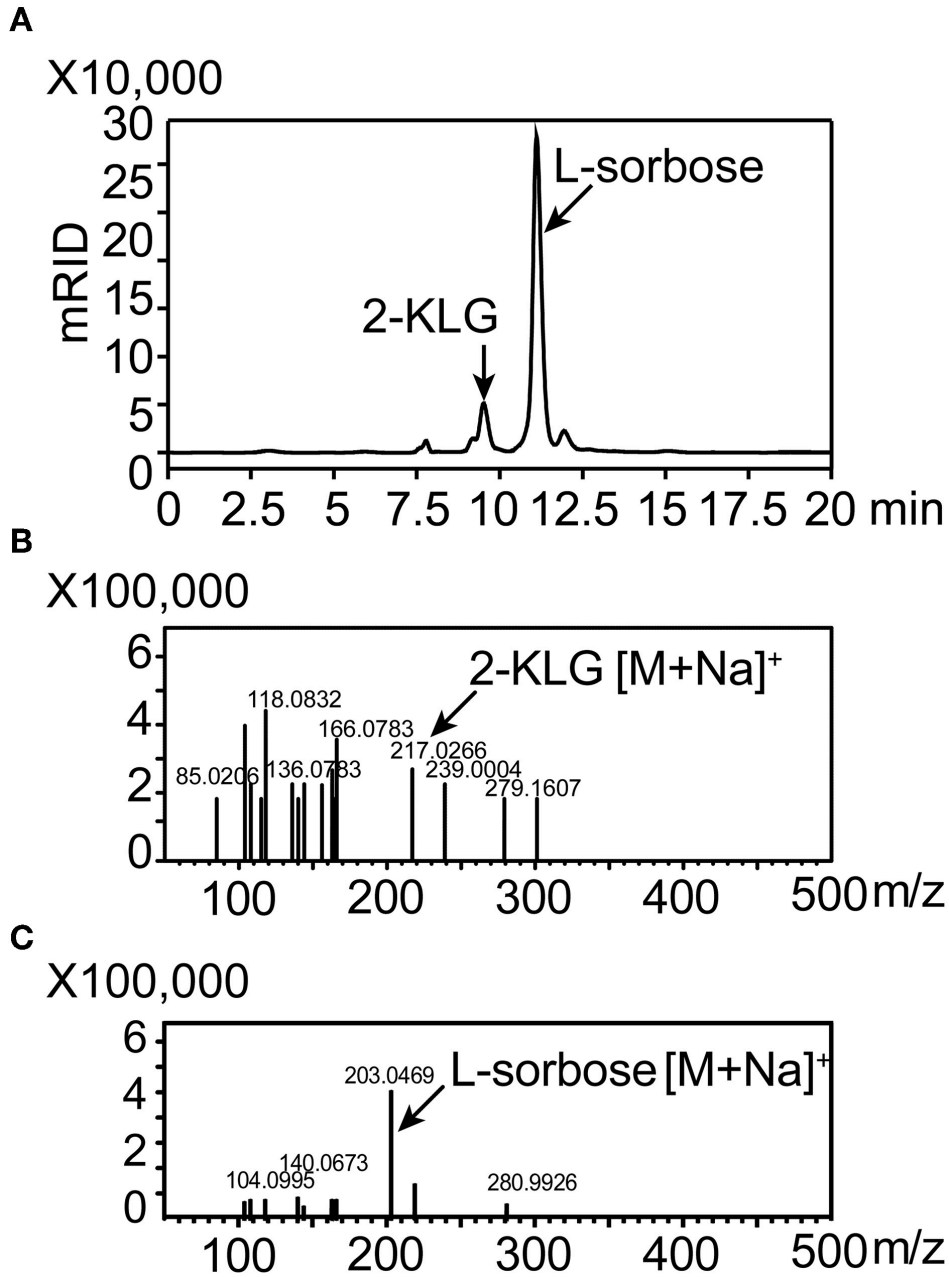
Identification of 2-KLG and L-sorbose fermentation by *Gluconobacter oxydans* WSH-004**. (A)** Identification of 2-KLG and L-sorbose in fermentation broth after 120 h of fermentation by *G. oxydans* WSH-004. **(B)** Identification of 2-KLG by LCMS-IT-TOF in positive ion mode. **(C)** Identification of L-sorbose by LCMS-IT-TOF in positive ion mode.

High-producing 2-KLG strains were obtained by screening soil and red tea fungus samples. Because living cells could hydrolyze fluorescein diacetate by expressing esterase, and the fluorescein released in this progress shared strong fluorescence signal in the channel of FITC. After enriching the strains for 48 h, FACS was performed with fluorescein diacetate to minimize human labor. Finally more than two hundreds 48-well MTPs and about 10 thousands strains were sorted. Potential strains were transferred to 250 mL shake flasks and the concentration of 2-KLG was verified by HPLC. A new *G. oxydans* WSH-004 strain, obtained from soil in local flower gardens, produced 2.5 g/L 2-KLG from D-sorbitol after a 120 h fermentation (Figure 6A). The product L-sorbose and 2-KLG were also verified by LCMS-IT-TOF (Figures 6B,C).

## Discussion

In previous studies, several wild *Gluconobacter* strains were reported to produce 2-KLG. *G. melanogenus* IFO 3292, IFO 3294, IFO 12257, and IFO 12258 were reported to produce 2-KLG <1 g/L (Sugisawa et al., 1990). Based on consumption of NADH when 2-KLG is converted to L-idonic acid by 2-KLG reductase, we established a rapid and accurate high-throughput screening method to measure the 2-KLG concentration in the fermentation broth. After several rounds of screening, we obtained a *G. oxydans* WSH-004 with 2.5 g/L of 2-KLG and accumulating about 45.2 g/L of L-sorbose from 50.0 g/L of D-sorbitol after 5 days fermentation. This result revealed that the rate-limiting step of the fermentation is the enzyme activity of sorbose dehydrogenase. Furthermore, compared with other 2-KLG producing strains, such as *G. oxydans* IFO 3293 that accumulated L-idonic acid from L-sorbose while producing 2-KLG, the newly obtained *G. oxydans* WSH-004 strain produced scarcely any side products. These results indicate that *G. oxydans* WSH-004 may be an appropriate one-step fermentation strain for producing 2-KLG.

In the past 40 years, many researchers have explored the production of 2-KLG by microorganisms (Zhou et al., 2012). Generally, two routes have been employed; the first is based on a conventional two-step fermentation process in which D-sorbitol is converted to L-sorbose by sorbitol dehydrogenase (SLDH), and L-sorbose is converted to 2-KLG by sorbose dehydrogenase (SDH) and sorbosone dehydrogenase (SNDH) (Gao et al., 2013); the second route is based on a novel two-step fermentation process in which D-glucose is converted to 2,5-diketo-D-gluonic acid (2,5-DKG) by three enzymes, and 2,5-DKG is then converted to 2-KLG by 2,5-DKG reductase (Sonoyama et al., 1982; Gao et al., 2014). Subsequently, one-step fermentation strains based on the above routes were also constructed and studied (Anderson et al., 1985; Gao et al., 2014). However, the yield of 2-KLG was too low to be competitive with the existing two-step fermentation process. Thus, further studies are required to solve this problem, and our present enzyme-based screening method will prove useful for this.

The conventional screening method for 2-KLG is based on co-cultivation of two bacterial strains (Zhao et al., 2008). In this method, *K. vulgare* colonies on plates are randomly selected for primary screening, used for fermentation with *Bacillus spp*. in glass tubes, then inoculated into flasks for further fermentation (Lu et al., 2005). This method is considered inefficient and time-consuming, which greatly limits the potential for one-step fermentation. A plate method has since been developed for rapid screening of *K. vulgare* mutants for enhanced 2-KLG production (Yang et al., 2017). This method greatly improved the probability of obtaining positive mutants. However, it cannot differentiate 2-KLG from other acidic metabolites in the medium and accurately probe the fermentation progress which requires pH control. Thus, selected mutants must be further confirmed by co-fermentation with *B. megaterium*. Our current enzyme-based high-throughput screening method solves this problem. The high substrate specificity of 2-KLG reductase distinguishes 2-KLG from other acidic metabolites in the medium, and the reaction is determined solely by the final 2-KLG concentration, and is not dependent on the intermediate product, the pH or fermentation progress. Furthermore, compared with other methods, this enzyme-based method can determine the concentration of 2-KLG in the fermentation broth more rapidly and precisely.

The enzyme-based high-throughput screening method developed herein has great potential for screening high-producing 2-KLG-producing strains in an efficient and accurate manner. However, there are still some shortages limiting its further applications. Compared with the pH based plate methods, this method requires an extra step for preparing 2-KLG reductase, which is a little complicated that the pH based method (Yang et al., 2017). Besides, both pH and 2-KLG reductase based high throughput screening strategies could only be compatible with multi-well plates. The screening amount is still limited to around 10^5^ strains for each round of screening. With the development of synthetic biology, more and more biosensor based methods have been established and employed in high throughput screening (Liu et al., 2017). Combination of biosensors and microfluidics strategies, more efficient screening strategies for 2-KLG producers with fluorescence activated cell sorting can be established and could further improve the screening amount up to 10^8^ strains per round.

## Data Availability Statement

The raw data supporting the conclusions of this manuscript will be made available by the authors, without undue reservation, to any qualified researcher.

## Author Contributions

YC, LL, and XS performed experiments and data analysis. YC and JZ wrote the manuscript and conceived the study. GD, JZ, and JC coordinated the project.

### Conflict of Interest

The authors declare that the research was conducted in the absence of any commercial or financial relationships that could be construed as a potential conflict of interest.
