# Supplementary figures and images for: High-Throughput Screening of a 2-Keto-L-Gulonic Acid-Producing Gluconobacter oxydans Strain Based on Related Dehydrogenases
Source: Front Bioeng Biotechnol. 2019 Dec 13;7:385. doi: 10.3389/fbioe.2019.00385 (PMC6923176; doi:10.3389/fbioe.2019.00385)

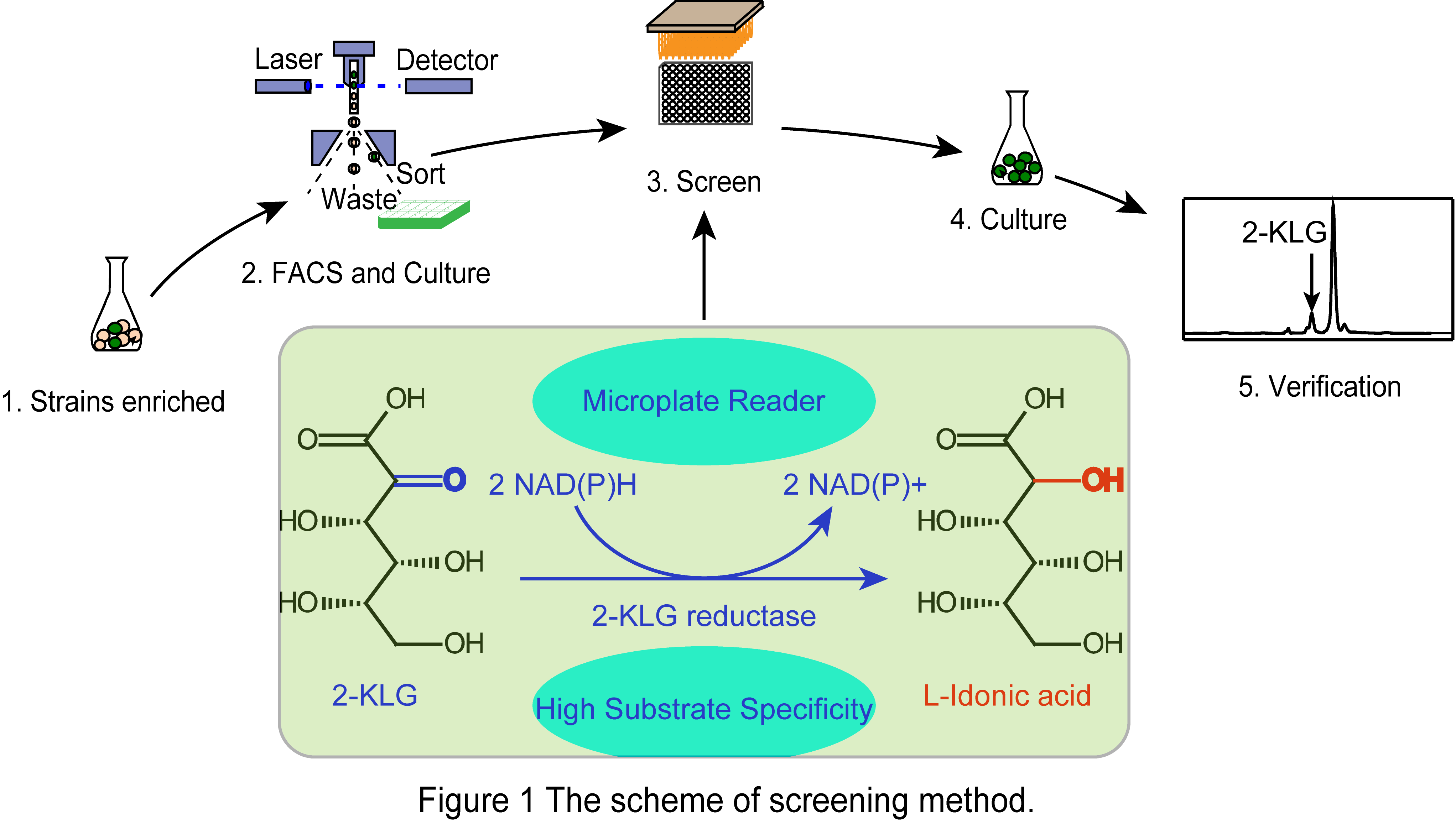

Supplement: Supplementary file 1 [file Image_1.tif]
